# Economic Analysis of Children’s Surgical Care in Low- and Middle-Income Countries: A Systematic Review and Analysis

**DOI:** 10.1371/journal.pone.0165480

**Published:** 2016-10-28

**Authors:** Anthony T. Saxton, Dan Poenaru, Doruk Ozgediz, Emmanuel A. Ameh, Diana Farmer, Emily R. Smith, Henry E. Rice

**Affiliations:** 1 Duke Global Health Institute and Duke University Medical Center, Durham, NC, United States of America; 2 McMaster Paediatric Surgery Research Collaborative, Dept. of Surgery, McMaster University, Hamilton, Canada; 3 Yale-New Haven Hospital, New Haven, CT, United States of America; 4 Department of Surgery, National Hospital, Abuja, Nigeria; 5 University of California-Davis Health System, Davis, CA, United States of America; University Medical Center of Princeton at Plainsboro, UNITED STATES

## Abstract

**Background:**

Understanding the economic value of health interventions is essential for policy makers to make informed resource allocation decisions. The objective of this systematic review was to summarize available information on the economic impact of children’s surgical care in low- and middle-income countries (LMICs).

**Methods:**

We searched MEDLINE (Pubmed), Embase, and Web of Science for relevant articles published between Jan. 1996 and Jan. 2015. We summarized reported cost information for individual interventions by country, including all costs, disability weights, health outcome measurements (most commonly disability-adjusted life years [DALYs] averted) and cost-effectiveness ratios (CERs). We calculated median CER as well as societal economic benefits (using a human capital approach) by procedure group across all studies. The methodological quality of each article was assessed using the Drummond checklist and the overall quality of evidence was summarized using a scale adapted from the Agency for Healthcare Research and Quality.

**Findings:**

We identified 86 articles that met inclusion criteria, spanning 36 groups of surgical interventions. The procedure group with the lowest median CER was inguinal hernia repair ($15/DALY). The procedure group with the highest median societal economic benefit was neurosurgical procedures ($58,977). We found a wide range of study quality, with only 35% of studies having a Drummond score ≥ 7.

**Interpretation:**

Our findings show that many areas of children’s surgical care are extremely cost-effective in LMICs, provide substantial societal benefits, and are an appropriate target for enhanced investment. Several areas, including inguinal hernia repair, trichiasis surgery, cleft lip and palate repair, circumcision, congenital heart surgery and orthopedic procedures, should be considered “Essential Pediatric Surgical Procedures” as they offer considerable economic value. However, there are major gaps in existing research quality and methodology which limit our current understanding of the economic value of surgical care.

## Introduction

Surgically treatable conditions constitute a large portion of the global burden of disease.[1] The consequences of inadequate surgical care in low and middle-income countries (LMICs) are especially dire among children, where many conditions result in lifelong disabilities or premature deaths.[2, 3] In recent years there has been a growing interest in surgery as a global health priority and recognition of its role in a functioning health care system in the World Bank’s *Disease Control Priorities in Developing Countries* and the *Lancet* Commission on Global Surgery.[4, 5]

Understanding the economic value of health interventions is essential for policy makers to make resource allocation decisions. Common types of economic evaluation includes cost analysis, which reports the monetary costs per procedure; cost-effectiveness analysis, which relates costs to a standardized health outcome such as infections averted or a quantifiable health metric; cost-benefit analysis, which describes patients’ willingness to pay for a treatment; and societal economic benefit, which quantifies the economic losses that will be reduced for an individual and society as a result of successful treatment of a condition. Cost-effectiveness analyses necessitates the use of health outcome metrics such as the Disability-Adjusted Life Year (DALY), which is a composite measure of years of life lost due to premature mortality and years lived with disability. DALY estimates require several assumptions, most importantly a disability weight (DW), which reflects the severity of a health state and ranges from 0 (perfect health) to 1 (equivalent to death). DALY and DW estimates have been thoroughly summarized in the Global Burden of Disease (GBD) studies and other reports,[6–9] although estimates for surgical care remain problematic for many reasons, such as the large number of diagnoses and procedures, complexity of surgical interventions, and limited number of DWs for conditions requiring surgical intervention[10]

Several recent studies have demonstrated that many areas of surgical care are cost-effective compared with other common health interventions, although these studies do not focus on care of children.[11–13] In this report, we performed a systematic review to describe the economic impact of children’s surgical care in LMICs with three goals: 1) to summarize published research on cost and cost-effectiveness of children’s surgical procedures, 2) to construct a reference table of reported disability weights for conditions requiring surgical procedures, and 3) to describe the societal economic benefit of surgical care for children.

## Methods

### Selection Criteria

For our systematic review, we followed the Preferred Reporting Items for Systematic Reviews and Meta-Analysis (PRISMA) guidelines[14] (see S1 Checklist) and registered the study with PROSPERO (registration number: CRD42015016059). Our search encompassed all English-language indexed articles published from January 1996-January 2015 that fulfilled each of the following criteria:

Surgery: studies analyzed surgical procedures, surgical facilities, or missions;
Pediatric population: recipients included children less than 18 years of age;
Economic analysis: studies reported care costs, DALYs averted, life years saved (LYS), quality-adjusted life years (QALYs) saved, health-adjusted life years (HALYs) gained, or societal economic benefit;
LMIC: care performed in a LMIC as defined by the World Bank as of February, 2015;[15]

### Data Sources

We conducted a search for eligible studies in MEDLINE (PubMed), Embase, and Web of Science on February 1, 2015. Database-specific indexing terms were used, with the full search listed in S1 Table. We collected additional articles outside the search by consulting experts and reviewing bibliographies of identified studies. We excluded systematic reviews or other non-primary data sources.

### Article Selection and Data Extraction

Two researchers (ATS and HER) screened abstracts of identified articles, and considered each full text for inclusion with disagreements settled by discussion. For each included report, we collected intervention type, population, cost, health outcomes, average cost-effectiveness ratio (CER), incremental cost-effectiveness ratio (ICER), and societal economic benefit. If a study did not report costs or health outcomes on a “per procedure” basis, we translated results to this format by dividing total amounts by reported procedure count. Interventions were organized into 36 procedure groups under 8 areas (cardiac surgery, otolaryngology/ear, nose, and throat surgery [ENT], general surgery, neurosurgery, ophthalmology, orthopedics, plastic and reconstructive surgery, and urology). We converted outcomes to 2015 United States dollars (USD) by using PPP conversion factors from the World Bank[16] and the Consumer Price Index Inflation calculator.[17] For studies spanning multiple years, we used the last year reported for conversion.

### Article Grading

To assess the quality of each report, we used the 10-point checklist of Drummond.[18] Two observers independently graded each article with disagreements resolved by discussion. To translate individual Drummond scores into a metric that captures overall evidence quality for each of the 36 procedure groups, we adapted a grading scale from the Agency for Healthcare Research and Quality (AHRQ)[19] to reflect overall evidence quality, quantity, and consistency by the following criteria:

High: high level of assurance that research findings are valid; no important disagreement exists across studies; includes at least one study with a Drummond score of 9–10 per procedure type.
Moderate: moderate level of assurance that findings are valid; little disagreement exists across studies; includes at least two studies with a score of 7–8.5 or one study with a score of 9–10 per procedure type.
Low: low level of assurance that findings are valid; category includes studies with conflicting results; less than two studies have scores that exceed 6.5 per procedure type.

### Disability Weight Reference Table

A reference table of disability weights for pediatric health conditions commonly requiring surgical procedures was created by compiling values from the GBD studies, with preference given to values reported by GBD 2013,[8] then GBD 2004,[7] and finally Poenaru et al.[20] There were no disability weights identified for cystic echinococcosis, inguinal hernia, or Ponseti clubfoot management in the aforementioned sources, so we reported disability weights published from articles in our review. For circumcision in Sub-Saharan Africa, we calculated a disability weight of 0.028 based on the assumption that a circumcision reduces HIV prevalence from 12% to 6%[21] with an average of 15.5 DALYs/case of HIV averted.[22]

### Cost-Effectiveness Assessment

We followed WHO-CHOICE guidelines [23] to assign each intervention into a cost-effectiveness category. Interventions with a maximum CER of less than that country’s gross domestic product (GDP) per capita as defined by the World Bank [24] were considered very cost-effective, interventions with a CER of less than three times the GDP per capita were cost-effective, and all others were not cost-effective. Using the upper and lower bounds of extracted CER (both average and incremental), we summarized the CER values reported from each article in the different procedure groups (using only those articles that used DALY as the health outcome). We also calculated median CERs for each procedure group using minimum and maximum values extracted from all articles that used DALYs as the base health metric. All statistical analyses were performed with STATA v14.1 (StataCorp LP).

### Societal Economic Benefit

To determine the societal economic benefit gained from surgical care, we used a human capital approach. This method equates the value of a human life to the discounted market value of the economic output produced by an individual over an expected lifetime, and is frequently used by WHO for cost analyses.[25] For articles that reported economic benefits, we converted results to a “per procedure” basis and reported the minimum and maximum values. For articles without information on societal economic benefit, we calculated this benefit by multiplying reported minimum and maximum DALYs averted, QALYs gained, HALYs gained, or LYS by purchasing power parity (PPP) estimates of gross national income (GNI)/capita from the World Bank.[24] For articles that used DALYs as the health outcome, we summarized the societal economic benefit for each procedure group by presenting the values for each individual intervention within that group, and also reported median values of each procedure.

## Results

### Literature Search

**Fig 1:**
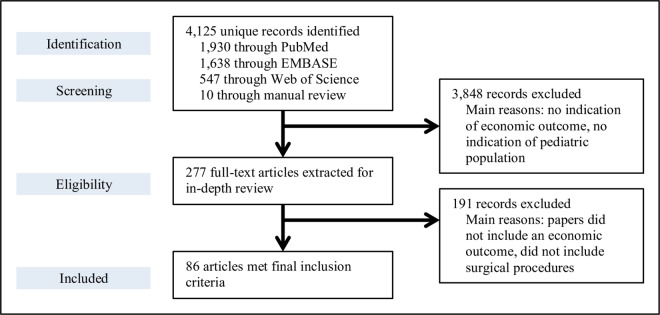
Preferred Reporting Items for Systematic Reviews and Meta-Analysis (PRISMA) flow diagram of the literature reviewing process on the economics of pediatric surgery in low- and middle-income countries. 4,125 articles were identified through our initial search, and altogether 86 studies met the full inclusion criteria to be included in our review.

We identified 4,125 articles through our initial search. Altogether, 86 studies met inclusion criteria (Fig 1),[26–111] with articles summarized into 36 different procedure groups.

### Article Grading and Disability Weights

The studies had a wide range of quality, with 65% of studies (n = 56) with Drummond scores 0–6.5, 19% (n = 16) with scores 7–8.5, and 16% (n = 14) with scores 9–10 (Table 1, individual scores in S2 Table). When summarized by procedure group, six had a high strength of evidence: inguinal hernia repair, trichiasis surgery, circumcision, cleft lip and palate repair, congenital heart defects, and orthopedic procedures. All other interventions had a moderate or low strength of evidence. Disability weights for pediatric surgical conditions identified from the GBD studies [7, 8] and others [20, 38, 72, 97] ranged from 0.005 for finger amputation, to 0.850 for posterior sagittal anorectoplasty (Table 2).

**Table 1:** Summary of scores using Drummond 10-point checklist and overall strength of evidence for 36 surgical interventions.

	Number of articles in Drummond score range^a^:				Strength of Evidence^b^
Intervention category	0–6.5	7–8.5	9–10	Total articles	
Cardiac surgery: congenital heart defects [85–94]	5	4	1	10	High
General Surgery: inguinal hernia repair [29, 35–39]	2	1	3	6	High
Ophthalmology: trichiasis surgery [54–56]	-	-	3	3	High
Orthopedics: various procedures [44,67–73]	5	2	1	8	High
PRS: cleft lip & palate [58–65]	3	2	1	6	High
Urology: circumcision [45–49]	2	-	3	5	High
Ophthalmology: cataract repair [56,80–83]	4	-	1	5	Moderate
Neurosurgery: hydrocephalus [66]	-	-	1	1	Moderate
Cardiac surgery: mediastinitis treatment [104]	-	1	-	1	Low
ENT: middle ear reconstruction [99]	1	-	-	1	Low
ENT: cochlear implantation [111]	1	-	-	1	Low
ENT: various procedures [44]	1	-	-	1	Low
General Surgery: anorectal reconstruction [28]	1	-	-	1	Low
General Surgery: appendectomy [29–32]	3	1	-	4	Low
General Surgery: Buruli ulcer [98]	1	-	-	1	Low
General Surgery: choledochal cyst excision [105]	1	-	-	1	Low
General Surgery: cystic echinococcosis [95–97]	2	1	-	3	Low
General Surgery: Hirschsprung’s repair [27]	1	-	-	1	Low
General Surgery: iliopsoas abscess [40]	1	-	-	1	Low
General Surgery: kidney transplantation [106]	1	-	-	1	Low
General Surgery: removal of ureteral stents [110]	1	-	-	1	Low
General Surgery: splenectomy [107, 108]	1	1	-	2	Low
General Surgery: various procedures [26,44,50]	2	1	-	3	Low
Neurosurgery: epilepsy procedures [78,79]	2	-	-	2	Low
Neurosurgery: frontoethmoidal meningoencephalocoele [51–53]	3	-	-	3	Low
Neurosurgery: various procedures [44,57]	2	-	-	2	Low
Ophthalmology: corneal ulcers [84]	1	-	-	1	Low
Ophthalmology: ocular trauma [42]	1	-	-	1	Low
Ophthalmology: retinopathy of prematurity [109]	-	1	-	1	Low
Orthopedics: amputation [103]	1	-	-	1	Low
Orthopedics: clubfoot treatment [33,34]	2	-	-	2	Low
Orthopedics: leg fractures [74–77]	3	1	-	4	Low
PRS: burn treatment [100–103]	4	-	-	4	Low
PRS: various procedures [43,44]	2	-	-	2	Low
Urology: genital reconstruction [41]	1	-	-	1	Low
Urology: various procedures [44]	1	-	-	1	Low

Abbreviations include: PRS plastic & reconstructive surgery; ENT ear, nose & throat.

^a^ Articles graded using the Drummond 10-point checklist to assess methodological quality of economic assessments.[18]

^b^ Strength of evidence determined by adapting the Evidence-Based Practice Centers of the Agency for Healthcare Research and Quality grading scale.[19] Ratings were assigned based on the quality, quantity, and consistency of the evidence in the literature.

**Table 2:** Disability weights for common pediatric surgical conditions.

Intervention	Disability weight^a^	Intervention	Disability weight^a^
**Cardiac Surgery**	** **	**Ophthalmology**	** **
Arterial switch operation [7]	0.323	Cataract repair [8]	0.031
Atrial septal defect [7]	0.323	Corneal ulcers [8]	0.031
Atrioventricular septal defect [7]	0.323	Laser treatment for retinopathy of prematurity [8]	
Blalock-Taussig shunt [7]	0.323		0.184
Coarctation of aorta [7]	0.323	Ocular trauma [7]	0.354
Fontan procedure [7]	0.323	Trachoma: Blindness [7]	0.570
Mediastinitis treatment [7]	0.323	Trachoma: Low vision [7]	0.170
Patent ductus arteriosus [7]	0.323	**Orthopedics**	** **
Tetralogy of Fallot [7]	0.323	Amputation: arm [8]	0.079
Totally anomalous pulmonary venous connection [7]		Amputation: thumb [8]	0.011
	0.323	Amputation: finger [8]	0.005
Ventricular septal defect [7]	0.323	Femoral shaft fractures [8]	0.042
**ENT**		Musculoskeletal injuries [8]	0.079
Chronic suppurative otitis media: middle ear reconstruction [8]		Open tibial injuries [8]	0.055
	0.158	Pediatric trauma: major surgical treatment [7]	
Cochlear implantation [8]	0.204		0.208
**General Surgery**		Pediatric trauma: minor procedures [8]	0.014
Appendectomy [7]	0.463	Ponseti clubfoot management [72]	0.231
Buruli ulcer [8]	0.051	Various orthopedic injury procedures [8]	
Choledochal cyst excision [8]	0.114		0.042
Cystic echinococcosis [97]	0.239	**Plastic and Reconstructive Surgery**	
Drainage of iliopsoas abscess [8]	0.114	Burns: superficial [8]	0.016
Hirschsprung’s repair: transanal endorectal pull-through [20]		Burns: partial thickness [8]	0.314
	0.720	Burns: full thickness [8]	0.314
Inguinal hernia repair [38]	0.300	Cleft lip [7]	0.082
Kidney transplantation [8]	0.547	Cleft palate [7]	0.216
Liver transplantation [7]	0.330	**Neurosurgery**	
Posterior sagittal anorectoplasty [7]	0.850	Epilepsy: anterior temporal lobe lobectomy [8]	
Removal of ureteral stents [7]	0.067		0.552
Splenectomy [8]	0.114	Epilepsy: corpus callosotomy [8]	0.552
**Urology**	** **	Frontoethmoidal meningoencephalocoele [8]	
Circumcision	0.028 ^b^		0.405
Genital reconstruction [8]	0.114	Hydrocephalus [20]	0.740

Abbreviations include: DALY disability-adjusted life years; DW disability weight

GBD Global Burden of Disease study; HIV human immunodeficiency virus.

^a^ Estimated DW values were assigned based on published values, with preference given to long-term over short-term values, and subtracting treated weights from untreated weights whenever both were listed. For conditions with multiple published DWs, preference was first given to the values from GBD 2013 study,[8] followed by the GBD 2004 update,[7] and then Poenaru et al.[20] For conditions without a DW from those sources, values published from other sources were used.

^b^ Calculated assuming circumcision intervention reduces HIV prevalence from 12% to 6% [21] with an average of 15.5 DALY/case of HIV averted [22] using the universal life expectancy of a 0 year old male with 3% discounting and age-weighting.

### Economic Outcomes

**Fig 2:**
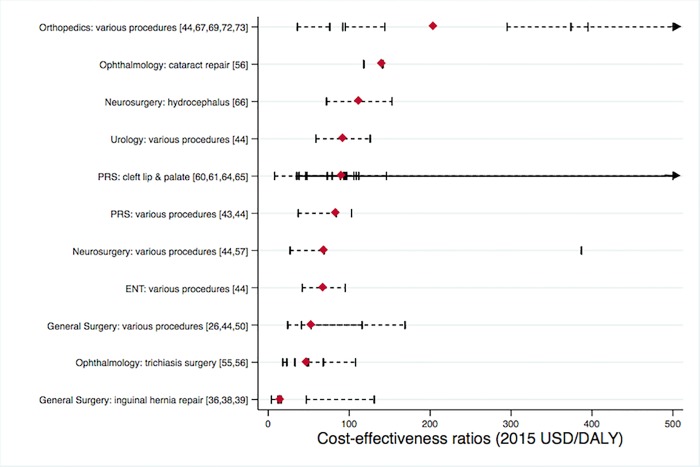
Cost-effectiveness (2015 USD/DALY) of 11 pediatric surgical intervention categories. Individual article references shown in brackets. Red diamonds represent median costs, black lines represent the range of values from each article. Abbreviations include: ENT ear, nose and throat; PRS plastic and reconstructive surgery; DALY disability-adjusted life year; USD United States dollar.

Of the 86 studies in our search, 84 reported cost data (detailed in S3 Table), 24 reported CER, and 2 reported societal economic benefit. In total, 53 procedural CERs were extracted (Table 3). There were 45 of the 53 CERs that reported DALY as the basis of health outcome (of which 36 used average costs and 9 used incremental costs for ICER), 3 reported QALY (2 average, 1 incremental), 3 used life-years saved or gained (2 average, 1 incremental), and 2 reported HALY (1 average, 1 incremental). The procedure with the lowest minimum CER was $4-14/DALY for inguinal hernia repair in Uganda.[39] All interventions were very cost-effective at each country’s GDP level by WHO standards. When summarized by procedure group, there were 12 procedures with CER information using DALYs as the health outcome. Eight of these had a median CER below $100, and 11 had a median CER below $1,000 (Fig 2). Inguinal hernia repair had the lowest median CER ($15), followed by trichiasis surgery ($48). Not shown in Fig 2 was cystic echinococcosis treatment, which had only one observation with a CER of $1,628/DALY.

**Table 3:** Cost-Effectiveness Ratios of Pediatric Surgical Procedures.

	Intervention	Location	Cost-effectiveness ratio, as reported^a^	Unit of outcome	Cost-effectiveness ratio, 2015 USD^b^
**Cardiac Surgery**					
Costa et al (2014) [94]	Atrial septal defects: percutaneous	Brazil	R$976	QALY, average	$553
			R$230,641	QALY, incremental	$123,550
	Atrial septal defects: surgical		R$631	QALY, average	$357
**Ear, Nose & Throat**					
Wu et al (2013) [44]	Various ENT procedures	Kenya	$42–95	DALY, average	$44–100
**General Surgery**					
Jha et al (1998) [29]	Appendectomy^e^	Guinea	$36	LYS, average	$58
	Inguinal hernia repair^e^		$74	LYS, average	$119
Gosselin et al (2006) [26]	Various general surgery	Sierra Leone	$33	DALY, average	$41
Shillcutt et al (2010) [36]	Inguinal hernia repair	Ghana	$11–15	DALY, incremental	$12–16
Wang et al (2012) [97]	Cystic echinococcosis	China	$1,469	DALY, average	$1,628
Ilbawi et al (2013) [50]	Various general surgery	Cameroon	$21–147	DALY, average	$24–169
Shillcutt et al (2013) [38]	Inguinal hernia repair	Ecuador	$44–123	DALY, incremental	$47–131
Wu et al (2013) [44]	Various general surgery	Kenya	$50–110	DALY, average	$53–116
Eeson et al (2015) [39]	Inguinal hernia repair	Uganda	$3–12	DALY, incremental	$4–14
**Neurosurgery**					
Warf et al (2011) [66]	Hydrocephalus	Uganda	$59–126	DALY, average	$72–153
Wu et al (2013) [44]	Various neurological operations	Kenya	$26–65	DALY, average	$27–69
Davis et al (2014) [57]	Various neurological operations	Guatemala	$385	DALY, average	$387
**Ophthalmology**					
Evans et al (1996) [54]	Trichiasis surgery	Burma	$59	HALY, average	$107
			$10	HALY, incremental	$18
Baltussen et al (2005) [55]	Trichiasis surgery	Africa	$13–17	DALY, average	$18–23
		Americas	$49	DALY, average	$68
		Mediterranean	$36–78	DALY, average	$50–108
		Southeast Asia	$24	DALY, average	$33
		Western Pacific	$35	DALY, average	$48
Baltussen et al (2012) [56]	Trichiasis surgery	Sub-Saharan Africa	$71–189	DALY, incremental	$86–230
	Trichiasis surgery		$71–90	DALY, average	$86–110
	Cataract repair		$116–117	DALY, incremental	$141–142
	Cataract repair		$116	DALY, average	$141
	Trichiasis surgery	Southeast Asia	$285–849	DALY, incremental	$347–1,034
	Trichiasis surgery		$285–374	DALY, average	$347–455
	Cataract repair		$97	DALY, incremental	$118
	Cataract repair		$97	DALY, average	$118
**Orthopedics**					
Grimes et al (2014) [67]	Various orthopedic procedures	Malawi	$92–139	DALY, average	$95–144
Gosselin et al (2008) [69]	Various orthopedic procedures	Cambodia	$77	DALY, average	$92
Gosselin et al (2011) [72]	Various orthopedic procedures	Nicaragua	$362	DALY, average	$395
		Dominican Repub.	$362	DALY, average	$395
		Haiti	$343	DALY, average	$374
Chen et al (2012) [73]	Various orthopedic procedures	Nicaragua	$270–719	DALY, incremental	$295–787
Wu et al (2013) [44]	Various orthopedic procedures	Kenya	$34–72	DALY, average	$36–76
**Plastic and Reconstructive Surgery**						
Corlew (2010) [60]	Cleft lip & palate	Nepal	$29–73	DALY, average	$35–89
Magee Jr. et al (2010) [61]	Cleft lip & palate	Vietnam	$7–850	DALY, average	$8–939
		Nicaragua	$66–1,828	DALY, average	$73–2,019
		Kenya	$96–1,193	DALY, average	$106–1,318
Moon et al (2012) [64]	Cleft lip & palate	Vietnam	$43–97	DALY, average	$47–106
Poenaru (2013) [65]	Cleft lip & palate	Africa	$72–134	DALY, average	$79–146
		Americas	$35–85	DALY, average	$38–93
		Europe	$35–89	DALY, average	$38–97
		Middle East	$42–100	DALY, average	$46–109
		Southeast Asia	$44–102	DALY, average	$48–112
		Western Pacific	$35–87	DALY, average	$38–95
Rattray et al (2013) [43]	Various reconstructive procedures	Cambodia	$99	DALY, average	$103
Wu et al (2013) [44]	Various reconstructive procedures	Kenya	$35–79	DALY, average	$37–84
**Urology**					
Binagwaho et al (2010) [45]	Circumcision	Rwanda	$334	LYG, incremental	$370
Wu et al (2013) [44]	Various urologic operations	Kenya	$56–119	DALY, average	$59–126

Abbreviations include: DALY disability-adjusted life-year; LYS life-year saved; LYG life-years gained; HALY health-adjusted life-year; QALY quality-adjusted life-year; USD United States dollar; Int. $ International dollars (a hypothetical currency unit with the same purchasing power parity that the U.S. dollar has in the United States at a given time).

^a^ All values presented on a per-procedure basis.

^b^ Values converted to USD using purchasing power parity conversion factors from the World Bank [16] and inflated to 2015 USD using the Consumer Price Index Inflation calculator.[17]

**Fig 3:**
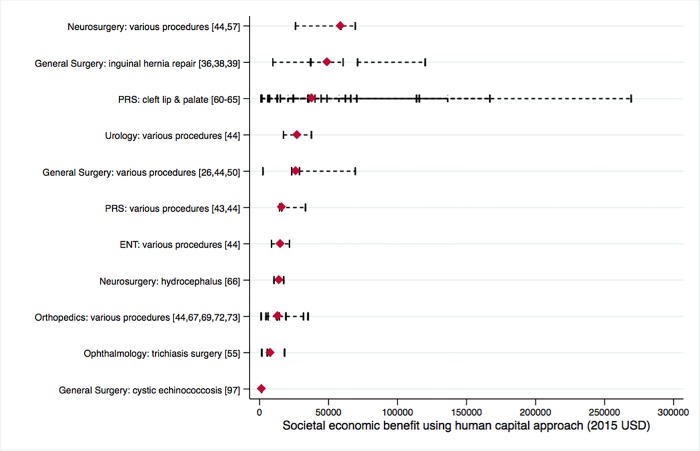
Societal economic benefit of 11 pediatric surgical intervention categories, measured using a human capital approach. Individual article references shown in brackets. Red diamonds represent median values, black bars represent the range of values from each article. Abbreviations include: ENT ear, nose and throat; PRS plastic and reconstructive surgery; USD United States dollars.

Many procedures were clinically effective as measured by averted DALYs, and most showed a substantial societal economic benefit (detailed in S4 Table). The intervention with the highest societal economic benefit was atrial septal defect repair in Brazil ($313,866). When summarized by procedure group, we were able to extract or calculate societal economic impact for 11 types of procedures based on articles that used DALYs as the health outcome. There were 9 out of those 11 procedure groups that had a median benefit greater than $10,000/procedure (Fig 3). Various neurosurgical procedures had the highest median economic benefit ($58,977), followed by inguinal hernia repair ($48,864).

## Discussion

Our review demonstrates that many areas of children’s surgical care in LMICs provide considerable economic value. These findings confirm the work of the *Lancet* Commission on Global Surgery and others, and suggest that surgical care is a valuable investment and should be incorporated as an essential component of a functioning health care system. Although we have reviewed the largest set of existing reports of children’s surgical care in LMICs, the limited range of economic analyses suggests that we have just examined the “tip of the iceberg” of pediatric surgical conditions.

As health care for children grows as a global health priority, this information should assist policy-makers for the development of funding priorities. However, as Baltussen et al. note, economic analyses should be used collectively with other criteria such as poverty reduction and severity of disease for a comprehensive approach to health care priority setting.[112] Use of a rights-based approach will ensure that a procedure that does not have a favorable cost-effectiveness or one that lacks adequate economic analysis is not excluded in provision of basic children’s health care services. Children’s surgical care should be thought of as an essential component of a “package of care,” as surgical care is an essential modality for the overall health care of children. Economic information should be taken into context by all local stakeholders, as local experts best understand the economic constraints within individual countries and the optimal methods to affect health care change.

Several particular areas of surgical care for children offer great economic value and are supported by high quality evidence. These should be considered as “Essential Children’s Surgical Procedures,” and include inguinal hernia repair, trichiasis surgery, cleft lip and palate repair, circumcision, congenital heart surgery, and orthopedic procedures. Our recommendations align with those of Mock et al.,[113] which prioritize surgical care based on health burden, success of a surgical intervention, and cost-effectiveness. We strongly advocate for increased investment in these “Essential Children’s Surgical Procedures” to improve health for children and gain economic benefit for a society. Many other procedures may potentially be quite cost-effective, although the quality of economic analysis is not as well developed, such as for Hirschsprung’s disease, anorectal reconstruction, appendectomy, clubfoot, abscess, meningoencephalocoele, trauma, hydrocephalus, epilepsy, and cataracts. Although our study strived to incorporate all available economic data on children’s surgical care, many areas still lack adequate economic analysis to make clear recommendations. For instance, we did not identify any economic studies for care of undescended testicles, typhoid perforation or intussusception. Although issues surrounding capacity and delivery of children’s surgical care are not addressed in our review, some procedures may be more appropriate for care and offer maximal economic benefits within different levels of a health system (i.e. district hospital, tertiary center).[114] Furthermore, innovative solutions such as task-sharing of surgical tasks to non-physician healthcare workers may be vital to maximize the economic value of surgical care for children.[115]

Moving forward, we encourage improvements in research methods to define the costs and value of surgical care. First, we advocate for refinement of the use of the DALY, DWs, and other output metrics for economic analysis of surgical treatments. Although a complete discussion of the limitations of the DALY and DW metrics for surgical care is beyond the scope of this review, they remains problematic for many reasons, such as the complexity of surgical care and procedures, identification of surgical component of care for health states requiring multidisciplinary care, or impact of surgical complications on health state outcomes.[10] Second, we support consistent use of WHO guidelines that define appropriate costs for economic analyses, which should reflect all customary costs for care in a public hospital setting, including infrastructure (capital costs, maintenance and utility costs, management overhead) and staff salaries, as well as indirect costs such as for travel or lost wages.[23] Third, we encourage the use of national datasets as they become available for economic analyses, such that variation between hospitals or researchers is minimized.

Existing disability weights incorporated into DALY estimates have been widely criticized for producing seemingly unrealistic values and have garnered concern regarding universal valuation for people from different geographic, cultural, or socioeconomic backgrounds.[116] Alternative measures of characterizing health states and the burden of conditions amenable to surgery include the epidemiological modeling strategy of Higashi et al.[117] Their model utilized inputs from GBD data to characterize the DALYs from congenital anomalies that could be potentially addressable from surgical intervention in LMIC, thereby estimating the excessive morbidity and mortality associated with these conditions. Coupling novel modeling strategies to enhance DALY estimations with comprehensive cost data will markedly improve economic analyses and facilitate scale-up modeling to help guide policy to improve surgical infrastructure.

Our study has several limitations inherent to economic analyses, including dependence on a wide range of assumptions as well as limited granularity of input data. Many articles report evidence from surgical missions and non-governmental organizations, which tend to underestimate or omit critical costs such as use of the facility, equipment depreciation, and overhead.[13] Also, the cost-effectiveness of an operation will vary by location and type of facility.[13] For example, costs associated with a surgical mission may be very different from a second-level hospital. Although all costs and CERs were converted and inflated to 2015 USD for consistency, our review included a wide timeframe stretching back to 1998, and some of the procedures described here may be outdated and no longer considered standard of care. Furthermore, many of the studies use different methods for age-weighting and discounting, which can impact the cost-effectiveness of a procedure. Some have argued that these practices are not appropriate,[118] and they have been subsequently omitted from the GBD 2010 study. Finally, we recognize limitations in assessment of the quality of surgical studies using the Drummond checklist, which is increasingly used for comparison of economic analyses across different health settings. Several items of the Drummond checklist are difficult to apply towards surgical care as noted by Marseille and Morshed,[119] such as reliance on the equal weighting of all criteria elements. However, the final score does allow for general comparison between studies. By translating quality assessments into “high, medium, or low” categories using AHRQ guidelines, we sought to translate these findings into commonly used categories.

Many of the CERs presented here from LMICs were based on average cost-effectiveness, which is most appropriate for scenarios where performing the intervention and doing nothing are the only options. This metric does not reflect the availability of alternate options with intermediate cost and effectiveness, and are problematic when comparing different treatments.[120] Analyses using ICERs would be needed to compare interventions that are mutually exclusive. In our analyses, all but one study used standard of usual care as the comparator for ICER calculations. However, ICERs are often not generalizable across different settings, because the “next best alternative” might vary by location. Thus, the results presented here are not intended to be used as an absolute standard for comparison between procedures, but rather to define a pattern from existing data to describe the economic benefits of pediatric surgical interventions in LMICs.

In summary, we found that many areas of surgical care for children are quite cost-effective and result in substantial economic benefits for LMICs. However, there are major gaps in existing research methodology which limit our understanding of the actual value of pediatric surgical care. Despite these limitations, it is clear that improved investment in surgical care in many setting is an extremely cost-effective approach to support health care for children, and should be considered as a key component of “packages of care” for children.

## Supporting Information

S1 Checklist PRISMA 2009 Checklist. (DOC)

S1 Table Database Search Terms. (PDF)

S2 Table Quality of Evidence using Drummon 10-Point Checklist. (PDF)

S3 Table Costs of Pediatric Surgical Procedures. (PDF)

S4 Table Health Outcomes and Societal Economic Benefit of Pediatric Surgical Procedures. (PDF)
