# Supplementary material for: Economic Analysis of Children’s Surgical Care in Low- and Middle-Income Countries: A Systematic Review and Analysis
Source: PLoS One. 2016 Oct 28;11(10):e0165480. doi: 10.1371/journal.pone.0165480 (PMC5085034; doi:10.1371/journal.pone.0165480)
Supplement: S1 Table — (PDF) [file pone.0165480.s002.pdf]

S1 Table: Database Search Terms

Database 1- Pubmed

Set Number	Search Terms for Pubmed	Results
1. Pediatrics	("Pediatrics"[Mesh] OR "Adolescent"[Mesh] OR "Child"[Mesh] OR "Infant"[Mesh] OR infant[tiab] OR neonat*[tiab] OR child[tiab] OR children[tiab] OR pediatric[tiab] OR adolescent*[tiab] OR teenage*[tiab] OR teen[tiab] OR teens[tiab] OR youth[tiab] OR youths[tiab] OR kid[tiab] OR kids[tiab])	3,233,386
2. Surgery	"Surgical Procedures, Operative"[Mesh] OR "surgery" [Subheading] OR “surgery”[tiab] OR “surgical”[tiab] OR "Hernia, Inguinal"[Mesh] OR "Testicular Hydrocele"[Mesh] OR "Cleft Lip"[Mesh] OR "Cleft Palate"[Mesh] OR "Cataract"[Mesh] OR "Anus, Imperforate"[Mesh]	3,457,007
3. LMIC	("Developing Countries"[Mesh] OR Africa[Mesh] OR "Central America"[Mesh] OR “American Samoa”[tiab] OR “Cambodia”[tiab] OR “China” [tiab] OR “Fiji”[tiab] OR “Indonesia”[tiab] OR “Kiribati”[tiab] OR “Korea”[tiab] OR “Lao”[tiab] OR “Malaysia”[tiab] OR “Marshall Islands”[tiab] OR “Micronesia”[tiab] OR “Mongolia”[tiab] OR “Myanmar”[tiab] OR “Palau”[tiab] OR “Papua New Guinea”[tiab] OR “Philippines”[tiab] OR “Samoa”[tiab] OR “Solomon Islands”[tiab] OR “Thailand”[tiab] OR “Timor-Leste”[tiab] OR “Tuvalu”[tiab] OR “Tonga”[tiab] OR “Vanuatu”[tiab] OR “Vietnam”[tiab] OR “Albania”[tiab] OR “Armenia”[tiab] OR “Azerbaijan”[tiab] OR “Belarus”[tiab] OR “Bosnia”[tiab] OR “Bulgaria”[tiab] OR “Georgia”[tiab] OR “Hungary”[tiab] OR “Kazakhstan”[tiab] OR “Kosovo”[tiab] OR “Kyrgyz Republic”[tiab] OR “Macedonia”[tiab] OR “Moldova”[tiab] OR “Montenegro”[tiab] OR “Romania”[tiab] OR “Serbia”[tiab] OR “Tajikistan”[tiab] OR “Turkey”[tiab] OR “Turkmenistan”[tiab] OR “Ukraine”[tiab] OR “Uzbekistan”[tiab] OR “Argentina”[tiab] OR “Belize”[tiab] OR “Bolivia”[tiab] OR “Brazil”[tiab] OR “Colombia”[tiab] OR “Costa Rica”[tiab] OR “Cuba”[tiab] OR “Dominica”[tiab] OR “Dominican Republic”[tiab] OR “Ecuador”[tiab] OR “El Salvador”[tiab] OR “Grenada”[tiab] OR “Guatemala”[tiab] OR “Guyana”[tiab] OR “Haiti”[tiab] OR “Honduras”[tiab] OR “Jamaica”[tiab] OR “Mexico”[tiab] OR “Nicaragua”[tiab] OR “Panama”[tiab] OR “Paraguay”[tiab] OR “Peru”[tiab] OR “St. Lucia”[tiab] OR “St. Vincent”[tiab] OR “Suriname”[tiab] OR “Venezuela”[tiab] OR “Algeria”[tiab] OR “Djibouti”[tiab] OR “Egypt”[tiab] OR “Iran”[tiab] OR “Iraq”[tiab] OR “Jordan”[tiab] OR “Lebanon”[tiab] OR “Libya”[tiab] OR “Morocco”[tiab] OR “Syria*”[tiab] OR “Tunisia”[tiab] OR “West Bank and Gaza”[tiab] OR “Yemen”[tiab] OR “Afghanistan”[tiab] OR “Bangladesh”[tiab] OR “Bhutan”[tiab] OR “India”[tiab] OR “Maldives”[tiab] OR “Nepal”[tiab] OR “Pakistan”[tiab] OR “Sri Lanka”[tiab] OR “Angola”[tiab] OR “Benin”[tiab] OR “Botswana”[tiab] OR “Burkina Faso”[tiab] OR “Burundi”[tiab] OR “Cameroon”[tiab] OR “Cabo Verde”[tiab] OR “Central African Republic”[tiab] OR “Chad”[tiab] OR “Comoros”[tiab] OR “Congo”[tiab] OR “Cote d’Ivoire”[tiab] OR “Ivory Coast”[tiab] OR “Eritrea”[tiab] OR “Ethiopia”[tiab] OR “Gabon”[tiab] OR “Gambia”[tiab] OR “Ghana”[tiab] OR “Guinea”[tiab] OR “Guinea-Bissau”[tiab] OR “Kenya”[tiab] OR “Lesotho”[tiab] OR “Liberia”[tiab] OR “Madagascar”[tiab] OR “Malawi”[tiab] OR “Mali”[tiab] OR “Mauritania”[tiab] OR “Mauritius”[tiab] OR “Mozambique”[tiab] OR “Namibia”[tiab] OR “Niger”[tiab] OR “Nigeria”[tiab] OR “Rwanda”[tiab] OR “São Tomé and Principe”[tiab] OR “Senegal”[tiab] OR “Seychelles”[tiab] OR “Sierra Leone”[tiab] OR “Somalia”[tiab] OR “South Africa”[tiab] OR “South Sudan”[tiab] OR “Sudan”[tiab] OR “Swaziland”[tiab] OR “Tanzania”[tiab] OR “Togo”[tiab] OR “Uganda”[tiab] OR “Zambia”[tiab] OR “Zimbabwe” OR "low resource"[tiab] OR "under-resourced"[tiab] OR "resource poor"[tiab] OR "under-developed"[tiab] OR "underdeveloped"[tiab] OR "developing country"[tiab] OR "developing countries"[tiab] OR "developing world"[tiab] OR "third world"[tiab] OR global[tiab] OR lmic[tiab] OR (low[tiab] AND middle[tiab] AND income[tiab])	1,005,679
4. STEP 1	1&2&3	23,833
5. Econ	("Costs and Cost Analysis"[Mesh]) OR "economics" [Subheading] OR “life year”[tiab] OR “life years”[tiab] OR “cost”[tiab] OR “costs”[tiab] OR “economic”[tiab] OR economics[tiab] OR “price”[tiab] OR “income”[tiab]	742,588
6. STEP 2	4&5	2,258

Database 2- Embase

Set Number	Search Terms for Embase	Results
1. Pediatrics	'pediatrics'/exp OR 'juvenile'/exp OR infant;ab,ti OR neonat*;ab,ti OR child;ab,ti OR children;ab,ti OR pediatric;ab,ti OR adolescent*;ab,ti OR teenage*;ab,ti OR teen;ab,ti OR teens;ab,ti OR youth;ab,ti OR youths;ab,ti OR kid;ab,ti OR kids;ab,ti	2,968,715
2. Surgery	'surgery'/exp OR 'surgery':lnk OR 'inguinal hernia'/exp OR 'hydrocele'/exp OR 'cleft lip'/exp OR 'cleft palate'/exp OR 'cataract'/exp OR 'anorectal malformation'/exp OR 'surgery':ab,ti OR 'surgical':ab,ti OR surgically:ab,ti OR 'imperforate anus':ab,ti OR 'cleft lip':ab,ti OR 'cleft palate':ab,ti OR hydrocele:ab,ti OR hernia:ab,ti OR cataract:ab,ti OR cataracts:ab,ti	4,633,248
3. LMIC	'developing country'/exp OR 'africa'/exp OR 'central america'/exp OR 'american samoa':ab,ti OR 'cambodia':ab,ti OR 'china':ab,ti OR 'fiji':ab,ti OR 'indonesia':ab,ti OR 'kiribati':ab,ti OR 'korea':ab,ti OR 'lao':ab,ti OR 'malaysia':ab,ti OR 'marshall islands':ab,ti OR 'micronesia':ab,ti OR 'mongolia':ab,ti OR 'myanmar':ab,ti OR 'palau':ab,ti OR 'papua new guinea':ab,ti OR 'philippines':ab,ti OR 'samoa':ab,ti OR 'solomon islands':ab,ti OR 'thailand':ab,ti OR 'timor-leste':ab,ti OR 'tuvalu':ab,ti OR 'tonga':ab,ti OR 'vanuatu':ab,ti OR 'vietnam':ab,ti OR 'albania':ab,ti OR 'armenia':ab,ti OR 'azerbaijan':ab,ti OR 'belarus':ab,ti OR 'bosnia':ab,ti OR 'bulgaria':ab,ti OR 'georgia':ab,ti OR 'hungary':ab,ti OR 'kazakhstan':ab,ti OR 'kosovo':ab,ti OR 'kyrgyz republic':ab,ti OR 'macedonia':ab,ti OR 'moldova':ab,ti OR 'montenegro':ab,ti OR 'romania':ab,ti OR 'serbia':ab,ti OR 'tajikistan':ab,ti OR 'turkey':ab,ti OR 'turkmenistan':ab,ti OR 'ukraine':ab,ti OR 'uzbekistan':ab,ti OR 'argentina':ab,ti OR 'belize':ab,ti OR 'bolivia':ab,ti OR 'brazil':ab,ti OR 'colombia':ab,ti OR 'costa rica':ab,ti OR 'cuba':ab,ti OR 'dominica':ab,ti OR 'dominican republic':ab,ti OR 'ecuador':ab,ti OR 'el salvador':ab,ti OR 'grenada':ab,ti OR 'guatemala':ab,ti OR 'guyana':ab,ti OR 'haiti':ab,ti OR 'honduras':ab,ti OR 'jamaica':ab,ti OR 'mexico':ab,ti OR 'nicaragua':ab,ti OR 'panama':ab,ti OR 'paraguay':ab,ti OR 'peru':ab,ti OR 'st. lucia':ab,ti OR 'st. vincent':ab,ti OR 'suriname':ab,ti OR 'venezuela':ab,ti OR 'algeria':ab,ti OR 'djibouti':ab,ti OR 'egypt':ab,ti OR 'iran':ab,ti OR 'iraq':ab,ti OR 'jordan':ab,ti OR 'lebanon':ab,ti OR 'libya':ab,ti OR 'morocco':ab,ti OR 'syria':ab,ti OR 'tunisia':ab,ti OR 'west bank and gaza':ab,ti OR 'yemen':ab,ti OR 'afghanistan':ab,ti OR 'bangladesh':ab,ti OR 'bhutan':ab,ti OR 'india':ab,ti OR 'maldives':ab,ti OR 'nepal':ab,ti OR 'pakistan':ab,ti OR 'sri lanka':ab,ti OR 'angola':ab,ti OR 'benin':ab,ti OR 'botswana':ab,ti OR 'burkina faso':ab,ti OR 'burundi':ab,ti OR 'cameroon':ab,ti OR 'cabo verde':ab,ti OR 'central african republic':ab,ti OR 'chad':ab,ti OR 'comoros':ab,ti OR 'congo':ab,ti OR 'cote ivoire':ab,ti OR 'ivory coast':ab,ti OR 'eritrea':ab,ti OR 'ethiopia':ab,ti OR 'gabon':ab,ti OR 'gambia':ab,ti OR 'ghana':ab,ti OR 'guinea':ab,ti OR 'guinea-bissau':ab,ti OR 'kenya':ab,ti OR 'lesotho':ab,ti OR 'liberia':ab,ti OR 'madagascar':ab,ti OR 'malawi':ab,ti OR 'mali':ab,ti OR 'mauritania':ab,ti OR 'mauritius':ab,ti OR 'mozambique':ab,ti OR 'namibia':ab,ti OR 'niger':ab,ti OR 'nigeria':ab,ti OR 'rwanda':ab,ti OR 'sao tome and principe':ab,ti OR 'senegal':ab,ti OR 'seychelles':ab,ti OR 'sierra leone':ab,ti OR 'somalia':ab,ti OR 'south africa':ab,ti OR 'south sudan':ab,ti OR 'sudan':ab,ti OR 'swaziland':ab,ti OR 'tanzania':ab,ti OR 'togo':ab,ti OR 'uganda':ab,ti OR 'zambia':ab,ti OR 'zimbabwe':ab,ti OR 'low resource':ab,ti OR 'under resourced':ab,ti OR 'resource poor':ab,ti OR 'under developed':ab,ti OR 'underdeveloped':ab,ti OR 'developing country':ab,ti OR 'developing countries':ab,ti OR 'developing world':ab,ti OR 'third world':ab,ti OR lmic:ab,ti OR (low:ab,ti AND middle:ab,ti AND income:ab,ti)	1,028,927
4. STEP 1	#1 AND #2 AND #3	19,208
5. Econ	'economic aspect'/exp OR 'economic aspect' OR 'life year':ab,ti OR 'life years':ab,ti OR cost:ab,ti OR costs:ab,ti OR economic:ab,ti OR economics:ab,ti OR price:ab,ti OR income:ab,ti OR 'value of a statistical life':ab,ti OR vsl:ab,ti OR daly:ab,ti OR financial:ab,ti OR fees:ab,ti OR fee:ab,ti OR expenditure:ab,ti OR expenditures:ab,ti OR expenses:ab,ti OR expense:ab,ti OR revenue:ab,ti OR salaries:ab,ti OR wage:ab,ti OR salary:ab,ti	1,543,164
6. STEP 2	#4 AND #5	2,457

Database 3- Web of Science

Set Number	Search Terms for Web of Science	Results
1. Pediatrics	'pediatrics' OR 'juvenile' OR infant OR neonat* child OR children OR pediatric OR adolescent OR teenage OR teen OR teens OR youth OR youths OR kid OR kids	1,716,232
2. Surgery	'surgery' OR 'inguinal hernia' OR 'hydrocele' OR 'cleft lip' OR 'cleft palate' OR 'cataract' OR 'anorectal malformation' OR 'surgery' OR 'surgical' OR surgically OR 'imperforate anus' OR 'cleft lip' OR 'cleft palate' OR hydrocele OR hernia OR cataract OR cataracts	1,148,764
3. LMIC	'developing country' OR 'africa' OR 'central america' OR 'american samoa' OR 'cambodia' OR 'china' OR 'fiji' OR 'indonesia' OR 'kiribati' OR 'korea' OR 'lao' OR 'malaysia' OR 'marshall islands' OR 'micronesia' OR 'mongolia' OR 'myanmar' OR 'palau' OR 'papua new guinea' OR 'philippines' OR 'samoa' OR 'solomon islands' OR 'thailand' OR 'timor-leste' OR 'tuvalu' OR 'tonga' OR 'vanuatu' OR 'vietnam' OR 'albania' OR 'armenia' OR 'azerbaijan' OR 'belarus' OR 'bosnia' OR 'bulgaria' OR 'georgia' OR 'hungary' OR 'kazakhstan' OR 'kosovo' OR 'kyrgyz republic' OR 'macedonia' OR 'moldova' OR 'montenegro' OR 'romania' OR 'serbia' OR 'tajikistan' OR 'turkey' OR 'turkmenistan' OR 'ukraine' OR 'uzbekistan' OR 'argentina' OR 'belize' OR 'bolivia' OR 'brazil' OR 'colombia' OR 'costa rica' OR 'cuba' OR 'dominica' OR 'dominican republic' OR 'ecuador' OR 'el salvador' OR 'grenada' OR 'guatemala' OR 'guyana' OR 'haiti' OR 'honduras' OR 'jamaica' OR 'mexico' OR 'nicaragua' OR 'panama' OR 'paraguay' OR 'peru' OR 'st. lucia' OR 'st. vincent' OR 'suriname' OR 'venezuela' OR 'algeria' OR 'djibouti' OR 'egypt' OR 'iran' OR 'iraq' OR 'jordan' OR 'lebanon' OR 'libya' OR 'morocco' OR 'syria' OR 'tunisia' OR 'west bank and gaza' OR 'yemen' OR 'afghanistan' OR 'bangladesh' OR 'bhutan' OR 'india' OR 'maldives' OR 'nepal' OR 'pakistan' OR 'sri lanka' OR 'angola' OR 'benin' OR 'botswana' OR 'burkina faso' OR 'burundi' OR 'cameroon' OR 'cabo verde' OR 'central african republic' OR 'chad' OR 'comoros' OR 'congo' OR 'cote ivoire' OR 'ivory coast' OR 'eritrea' OR 'ethiopia' OR 'gabon' OR 'gambia' OR 'ghana' OR 'guinea' OR 'guinea-bissau' OR 'kenya' OR 'lesotho' OR 'liberia' OR 'madagascar' OR 'malawi' OR 'mali' OR 'mauritania' OR 'mauritius' OR 'mozambique' OR 'namibia' OR 'niger' OR 'nigeria' OR 'rwanda' OR 'sao tome and principe' OR 'senegal' OR 'seychelles' OR 'sierra leone' OR 'somalia' OR 'south africa' OR 'south sudan' OR 'sudan' OR 'swaziland' OR 'tanzania' OR 'togo' OR 'uganda' OR 'zambia' OR 'zimbabwe' OR 'low resource' OR 'under resourced' OR 'resource poor' OR 'under developed' OR 'underdeveloped' OR 'developing country' OR 'developing countries' OR 'developing world' OR 'third world' OR lmic	2,499,993
4. STEP 1	#1 AND #2 AND #3	5,082
5. Econ	'economic aspect' OR 'economic aspect' OR 'life year' OR 'life years' OR cost OR costs OR economic OR economics OR price OR income OR 'value of a statistical life' OR vsl OR daly OR financial OR fees OR fee OR expenditure OR expenditures OR expenses OR expense OR revenue OR salaries OR wage OR salary	2,078,342
6. STEP 2	#4 AND #5	892
