# Supplementary material for: Economic Analysis of Children’s Surgical Care in Low- and Middle-Income Countries: A Systematic Review and Analysis
Source: PLoS One. 2016 Oct 28;11(10):e0165480. doi: 10.1371/journal.pone.0165480 (PMC5085034; doi:10.1371/journal.pone.0165480)
Supplement: S2 Table — (PDF) [file pone.0165480.s003.pdf]

S2 Table Quality of Evidence using Drummond 10-Point Checklist

	Drummond checklist question													
	**1**	**2**	**3**	**4**	**5a**	**5b**	**6a**	**6b**	**7a**	**7b**	**8**	**9**	**10**	**Summary Score**
Evans et al. (2014)	Y	N	N	Y	Y	N	Y	N	Y	N	N	N	Y	4.5
Costa et al. (2014)	Y	Y	Y	Y	Y	Y	Y	Y	N	N	Y	Y	Y	9
Eeson et al. (2015)	Y	Y	Y	Y	Y	Y	Y	Y	Y	Y	Y	Y	Y	10
Davis et al. (2014)	Y	N	Y	Y	Y	Y	Y	Y	N/A	Y	N	N	Y	6.5
Ramachandran et al. (2013)	Y	N	N	Y	Y	N	Y	N	N/A	N	N	Y	N	4
Ilbawi et al. (2013)	Y	N	N	Y	Y	Y	Y	Y	N	N	N	N	Y	5
Kerr et al. (2012)	Y	N	N	N	Y	N	Y	N	Y	N	N	Y	Y	4.5
Banu et al. (2013)	N	N	N	Y	Y	N	N	N	N	N	N	N	N	1.5
Zhu et al. (2013)	Y	N	Y	Y	Y	N	Y	N	Y	N	N	N	N	4.5
Wu et al. (2013)	Y	N	N	Y	Y	Y	Y	Y	N	N	N	Y	Y	6
Poenaru (2013)	Y	N	N	Y	Y	Y	Y	Y	N	Y	N	Y	Y	6.5
Shillcutt et al. (2013)	Y	Y	Y	Y	Y	Y	Y	Y	Y	Y	Y	Y	Y	10
Moon et al. (2012)	Y	Y	Y	Y	Y	Y	Y	Y	Y	Y	N	Y	Y	9
Dave et al. (2012)	Y	Y	N	Y	Y	Y	Y	Y	Y	N	Y	N	Y	7.5
Chen et al. (2012)	Y	Y	Y	N	Y	Y	Y	Y	N/A	Y	Y	N	Y	7.5
Hughes et al. (2012)	Y	Y	Y	Y	Y	Y	Y	Y	Y	Y	Y	N	Y	9
Alkire et al. (2011)	Y	Y	N	N	N	Y	N	Y	Y	Y	Y	Y	Y	7
Gosselin et al. (2011)	Y	N	N	N	Y	Y	N	Y	N/A	Y	N	N	Y	4
Shillcutt et al. (2010)	Y	Y	N	Y	Y	Y	Y	Y	Y	Y	Y	Y	Y	9
Gosselin et al. (2008)	Y	Y	Y	Y	Y	Y	Y	Y	N/A	Y	Y	N	Y	8.5
Warf et al. (2011)	Y	Y	Y	Y	Y	Y	Y	Y	Y	Y	N	Y	Y	9
Allorto et al. (2011)	Y	Y	N	N	Y	N	Y	N	N	N	N	N	N	3
Mughal et al. (2011)	Y	Y	N	N	Y	N	Y	N	N/A	N	N	Y	Y	5
Sadoh et al. (2011)	Y	N	N	N	N	N	N	N	N/A	N	N	N	N	1
Zeinaloo et al. (2008)	Y	Y	Y	N	Y	N	Y	N	N/A	N	N	Y	N	5
Chen et al. (2007)	Y	Y	Y	Y	Y	N	Y	N	N	N	N	N	Y	6
Pratap et al. (2007)	Y	Y	Y	N	N	N	Y	N	N	N	N	Y	Y	5.5
Prajna et al. (2007)	Y	N	Y	Y	Y	N	Y	N	N/A	N	N	Y	Y	6
Tollefson et al. (2006)	Y	N	Y	Y	Y	N	Y	N	N/A	N	N	N	Y	5
Vida et al. (2006)	Y	Y	Y	Y	Y	N	Y	N	N	N	N	Y	Y	7
Gangopadhyay et al. (2006)	Y	Y	Y	N	Y	N	Y	N	Y	N	N	N	Y	5.5
Nursal et al. (2003)	Y	N	Y	Y	N	N	N	N	N	N	N	N	Y	4
Afaq et al. (2002)	Y	Y	Y	N	Y	N	Y	N	N	N	N	N	N	4
Manji et al. (2000)	Y	N	Y	N	Y	N	Y	N	N	N	N	N	Y	4
Asiedu et al. (1998)	Y	Y	N	N	Y	N	Y	N	Y	N	N	N	Y	4.5
Rattray et al. (2013)	Y	N	N	Y	Y	Y	Y	Y	N/A	Y	N	N	Y	5.5
Liu et al. (2012)	Y	Y	Y	Y	Y	N	Y	N	N	N	N	Y	Y	7

	Drummond checklist question													
	**1**	**2**	**3**	**4**	**5a**	**5b**	**6a**	**6b**	**7a**	**7b**	**8**	**9**	**10**	**Summary Score**
Baltussen et al. (2012)	Y	Y	N	Y	Y	Y	Y	Y	Y	Y	Y	Y	Y	9
Hsu et al. (2009)	Y	Y	Y	N	Y	N	Y	N	N	N	N	N	Y	5
Talwar et al. (2008)	N	N	N	N	Y	N	Y	N	N	N	N	Y	N	2
Baltussen et al. (2005)	Y	Y	Y	Y	Y	Y	Y	Y	Y	Y	N	Y	Y	9
Paudel et al. (2003)	N	N	Y	Y	Y	N	Y	N	N/A	N	N	Y	N	4
Perveen et al. (2014)	N	Y	Y	N	Y	N	N	N	N	N	N	N	N	2.5
Agarwal et al. (2010)	Y	N	N	N	Y	N	Y	N	N/A	N	N	N	N	2
Adoga et al. (2010)	Y	Y	N	Y	Y	N	Y	N	N	N	N	N	Y	5
Vida et al. (2007)	Y	Y	Y	Y	Y	N	Y	N	N	N	N	Y	Y	7
Vida et al. (2006)	Y	Y	Y	Y	Y	N	Y	N	N	N	N	Y	Y	7
Osuigwe et al. (2006)	Y	Y	Y	N	Y	N	N	N	N	N	N	N	Y	4.5
Dada et al. (2009)	Y	Y	Y	N	Y	N	N	N	N	N	N	N	Y	4.5
Fandino-Franky et al. (2000)	Y	N	Y	Y	Y	N	Y	N	Y	N	N	Y	Y	6.5
Gurses et al. (2003)	Y	Y	Y	Y	Y	N	Y	N	N	N	N	Y	N	6
Gogate et al. (2010)	Y	Y	N	Y	Y	N	Y	N	N	N	N	Y	Y	6
Grimes et al. (2014)	Y	Y	Y	Y	Y	Y	Y	Y	Y	Y	N	Y	Y	9
Gosselin et al. (2009)	Y	Y	Y	Y	Y	N	Y	N	N	N	Y	Y	Y	8
Panni et al. (2011)	Y	Y	Y	Y	Y	N	Y	N	Y	N	N	Y	Y	7.5
Reddy et al. (2009)	Y	N	Y	Y	Y	N	Y	N	N/A	N	N	N	N	4
Alinia et al. (2013)	Y	N	N	Y	N	N	N	N	N/A	N	N	N	Y	3
Gollogly et al. (2008)	Y	Y	N	Y	N	N	N	N	N	N	N	Y	N	4
Ekenze et al. (2007)	N	N	Y	N	Y	N	Y	N	N	N	N	Y	Y	4
Yang et al. (2010)	Y	Y	Y	Y	Y	N	Y	N	N	N	N	N	Y	6
Gray et al. (2007)	Y	Y	Y	N	Y	Y	Y	Y	N	N	N	N	Y	6
Rao et al. (2000)	Y	Y	Y	Y	Y	N	Y	N	N	N	N	N	Y	6
Hong et al. (2008)	N	N	Y	N	N	N	N	N	N	N	N	Y	Y	3
Utpal (2005)	Y	Y	Y	N	Y	N	Y	N	N/A	N	N	N	N	4
Binagwaho et al. (2010)	Y	Y	Y	Y	Y	Y	Y	Y	Y	Y	Y	Y	Y	10
Tumwesigyea et al. (2013)	Y	Y	Y	Y	Y	Y	Y	Y	N/A	Y	Y	Y	Y	9.5
Vasquez et al. (2009)	N	Y	Y	N	Y	N	Y	N	N	N	N	Y	Y	5
Murithi et al. (2008)	N	N	Y	N	Y	N	Y	N	N	N	N	N	Y	3
Akinyoola et al. (2011)	Y	Y	Y	Y	Y	N	Y	N	N	N	N	Y	N	6
Adegbehingbe et al. (2010)	N	Y	Y	N	Y	N	N	N	N	N	N	N	Y	3.5
Hodges et al. (2000)	N	N	Y	N	Y	N	Y	N	N/A	N	N	N	N	2
Gradin et al. (2012)	Y	Y	Y	N	Y	N	Y	N	N	N	N	N	Y	5
Wu et al. (2014)	Y	Y	Y	N	Y	N	Y	N	N	N	N	Y	Y	6

	Drummond checklist question													
	**1**	**2**	**3**	**4**	**5a**	**5b**	**6a**	**6b**	**7a**	**7b**	**8**	**9**	**10**	**Summary Score**
Chen et al. (2009)	Y	Y	Y	N	Y	N	Y	N	N	N	N	Y	Y	6
Sancaktutar et al. (2012)	Y	N	Y	N	Y	N	Y	N	N	Y	N	N	N	3.5
Roux et al. (2007)	N	Y	Y	N	Y	N	Y	N	N	N	N	N	N	3
Padhi et al. (2007)	Y	Y	Y	N	Y	N	Y	N	N	N	N	N	Y	5
Hamamci et al. (2002)	Y	Y	Y	Y	Y	N	Y	N	N	N	N	Y	Y	7
Njeuhmeli et al. (2011)	Y	Y	Y	Y	Y	Y	Y	Y	N/A	Y	Y	Y	Y	9.5
Magee et al. (2010)	Y	Y	Y	N	Y	Y	Y	Y	N/A	Y	N	Y	Y	7.5
Gosselin et al. (2006)	Y	Y	Y	Y	Y	Y	Y	Y	N/A	Y	N	N	Y	7.5
Corlew (2010)	Y	Y	Y	Y	Y	Y	Y	Y	N/A	Y	N	Y	Y	8.5
Jha et al. (1998)	Y	Y	Y	Y	Y	Y	Y	Y	Y	Y	N	N	Y	8
Evans et al. (1996)	Y	Y	Y	Y	Y	Y	Y	Y	Y	Y	Y	Y	Y	10
Oucheng et al. (2010)	N	N	Y	N	Y	N	N	N	N	N	N	N	N	1.5
Wang et al. (2012)	Y	Y	Y	N	Y	Y	Y	Y	N	N	N	Y	Y	7

Drummond checklist questions are: 1= Was a well-defined question posed in answerable form? 2= Was a comprehensive description of the competing alternatives given (i.e. can you tell who did what to whom, where, and how often)? 3=Was the effectiveness of the program or services established?; 4=Were all the important and relevant costs and consequences for each alternative identified?;5a=Were costs measured accurately in appropriate physical units (e.g. hours of nursing time, number of physician visits, lost work-days, gained life years)? 5b=Were consequences measured accurately in appropriate physical units (e.g. hours of nursing time, number of physician visits, lost work-days, gained life years)? 6a=Were the cost valued credibly?; 6b=Were the consequences valued credibly?;7a=Were costs adjusted for differential timing? 7b=Were consequences adjusted for differential timing? 8=Was an incremental analysis of costs and consequences of alternatives done?; 9=Was allowance made for uncertainty in the estimates of costs and consequences?; 10=Did the presentation and discussion of study results include all issues of concern to users?
