# Supplementary material for: Economic Analysis of Children’s Surgical Care in Low- and Middle-Income Countries: A Systematic Review and Analysis
Source: PLoS One. 2016 Oct 28;11(10):e0165480. doi: 10.1371/journal.pone.0165480 (PMC5085034; doi:10.1371/journal.pone.0165480)
Supplement: S3 Table — (PDF) [file pone.0165480.s004.pdf]

S3 Table. Costs of Pediatric Surgical Procedures

	Intervention	Location	Currency	Cost per outcome, as reported[a]	Cost per outcome, 2015 USD[b]
**Cardiac Surgery**					
Vida et al (2006)[85]	Ventricular septal defect	Guatemala	2005 USD	$3,787	$4,611
Vida et al (2006)[86]	Atrial septal defect: percutaneous occlusion	Guatemala	2005 USD	$4,521	$5,505
	Atrial septal defect: surgical closure			$3,330	$4,054
Vida et al (2007)[104]	Mediastinitis treatment: delayed sternal closure	Guatemala	2005 USD	$9,612	$11,704
	Mediastinitis treatment: primary sternal closure			$2,467	$3,004
Talwar et al (2008)[87]	Atrial septal defect	India	2007 Indian Rupee	₹21,000-70,000	$1,844-6,147
	Ventricular septal defect			₹23,000-75,000	$2,020-6,586
	Tetralogy of Fallot			₹24,000-80,000	$2,107-7,025
	Atrioventricular septal defect			₹55,000	$4,830
	Fontan procedure			₹24,000-80,000	$2,107-7,025
	Arterial switch operation			₹27,000-90,000	$2,371-7,903
	Patent ductus arteriosus			₹6,000-18,000	$527-1,581
	Coarctation of aorta			₹7,000-22,000	$615-1,932
	Blalock-Taussig shunt			₹6,000-20,000	$526-1,756
	Totally anomalous pulmonary venous connection			₹65,000	$5,708
Zeinaloo et al (2008)[88]	Patent ductus arteriosus	Iran	2005 USD	$1,697	$2,066
	Atrial septal defect			$2,739	$3,335
	Ventricular septal defect			$2,637	$3,211
Chen et al (2009)[89]	Patent ductus arteriosus: surgical closure	China	2007 Chinese Yuan	¥13,841	$4,882
	Patent ductus arteriosus: transcatheter Amplatzer occlusion			¥18,709	$6,598
Mughal et al (2011)[90]	Open heart surgery	Pakistan	2010 USD	$933	$1,018
	Closed heart surgery			$150	$163
	Angiographic interventions			$823	$897
Panni et al (2011)[91]	Congenital heart defects: age <1 year	Pakistan	2009 USD	$4,054	$4,494
	Congenital heart defects: age 1-5 years			$5,548	$6,150
Sadoh et al (2011)[92]	Patent ductus arteriosus	Nigeria	2009 USD	$4,000	$4,434
Liu et al (2012)[93]	Ventricular septal defect: percutaneous closure	China	2011 USD	$3,870	$4,091
	Ventricular septal defect: surgical closure			$4,583	$4,845
Costa et al (2014)[94]	Atrial septal defects: percutaneous closure	Brazil	2011 Brazil Real	R$19,267	$13,800
	Atrial septal defects: surgical closure			R$12,431	$8,904
**ENT**					
Adoga et al (2010)[99]	Chronic suppurative otitis media: middle ear reconstruction	Nigeria	2008 USD	$463	$511
Kerr et al (2012)[111]	Cochlear implantation	South Africa	2010 S. African Rand	R298,961	$68,952
Wu et al (2013)[44]	Various ENT procedures	Kenya	2011 USD	$354	$373
**General Surgery**					
Asiedu et al (1998)[98]	Buruli ulcer	Ghana	1996 USD	$659-967	$998-1,465
Jha et al (1998)29	Appendectomy[e]	Guinea	1994 USD	$67	$107
	Inguinal hernia repair[e]			$53	$85
Afaq et al (2002)[40]	Drainage of iliopsoas abscess	Nepal	2000 USD	$40	$55
Hamamci et al (2002)[107]	Laparoscopic splenectomy	Turkey	2000 USD	$2,064 ($1,767-2,687)	$2,850 ($2,440-3,711)
	Open splenectomy			$1,664 ($848-3,152)	$2,298 ($1,171-4,353)
Paudel et al (2003)[30]	Appendectomy	Nepal	2003 Nepalese Rupee	Rs 2,485 (Rs 1,372-4,500)	$178 ($98-322)
Utpal (2005)[31]	Laparoscopic appendectomy	India	2005 Indian Rupee	₹925	$86
	Open appendectomy			₹923	$86
Gangopadhyay et al (2006)[28]	Posterior sagittal anorectoplasty: multi-stage procedure	India	2003 Indian Rupee	₹19,000	$1,844
	Posterior sagittal anorectoplasty: single stage procedure			₹5,000	$485
Gosselin et al (2006)[26]	Various general surgery	Sierra Leone	2004 USD	$43	$54
Osuigwe et al (2006)[35]	Inguinal hernia repair	Nigeria	2003 USD	$31-43	$40-56
Chen et al (2007)[108]	Laparoscopic splenectomy	China	2006 USD	$871	$1,028
	Open splenectomy			$783	$924
Pratap et al (2007)27	Hirschsprung's repair: transanal endorectal pull-through	Nepal	2005 Nepalese Rupee	Rs5,000 (Rs3,500-8,000)	$344 ($241-550)
Hong et al (2008)[105]	Laparoscopic choledochal cyst excision	China	2007 Chinese Yuan	¥19,167 (¥12,600-34,538)	$6,760 ($4,444-12,181)
Vasquez et al (2009)[95]	Cystic echinococcosis	Peru	2005 USD	$831 ($564-1,610)	$1,012 ($687-1,960)
Shillcutt et al (2010)[36]	Inguinal hernia repair	Ghana	2008 USD	$120	$132
Yang et al (2010)[96]	Cystic echinococcosis	China	2002 USD	$208-540	$275-714
Sancaktutar et al (2012)[110]	Removal of ureteral stents	Turkey	2010 USD	$1,225 ($330-3,800)	$1,336 ($360-4,144)
Wang et al (2012)97	Cystic echinococcosis	China	2008 USD	$1,493	$1,644
Banu et al (2013)[37]	Inguinal hernia repair	Bangladesh	2011 USD	$21	$22
Ilbawi et al (2013)[50]	Various general surgery	Cameroon	2007 USD	$215	$246
Ramachandran et al (2013)[106]	Kidney transplantation	India	2011 USD	$7,881 ($2,151-23,792)	$8,332 ($2,274-25,153)
Shillcutt et al (2013)[38]	Inguinal hernia repair	Ecuador	2011 USD	$499	$526
Wu et al (2013)[44]	Various general surgery	Kenya	2011 USD	$1,156	$1,218
Wu et al (2014)[32]	Appendectomy: single-site laparoscopic procedure	China	2013 Chinese Yuan	¥12,282	$3,643
	Appendectomy: 3-port laparoscopic procedure			¥12,187	$3,615
Eeson et al (2015)[39]	Inguinal hernia repair	Uganda	2010 USD	$87	$95

	Intervention	Location	Currency	Cost per outcome, as reported[a]	Cost per outcome, 2015 USD[b]
**Neurosurgery**					
Fandino-Franky et al (2000)[78]	Epilepsy: corpus callosotomy	Colombia	1999 USD	$3,137-3,995	$4,478-5,702
Rao et al (2000)[79]	Epilepsy: anterior temporal lobe lobectomy	India	1998 USD	$1,200	$1,751
Roux et al (2007)[51]	Frontoethmoidal meningoencephalocoele repair	Cambodia	2006 USD	$380	$448
Gollogly et al (2008)[52]	Frontoethmoidal meningoencephalocoele repair	Cambodia	2007 USD	$300-500	$344-573
Oucheng et al (2010)[53]	Frontoethmoidal meningoencephalocoele repair	Cambodia	2009 USD	$380	$421
Warf et al (2011)[66]	Hydrocephalus	Uganda	2005 USD	$746	$905
Wu et al (2013)[44]	Various neurological operations	Kenya	2011 USD	$608	$641
Davis et al (2014)[57]	Various neurological operations	Guatemala	2014 USD	$3,127	$3,131
**Ophthalmology**					
Evans et al (1996)[54]	Trichiasis surgery	Burma	1990 USD	$1,099	$1,993
Baltussen et al (2005)[55]	Trichiasis surgery	Africa	2000 Int. $	$19	$26
		Americas		$19	$26
		Mediterranean		$19	$26
		Southeast Asia		$19	$26
		Western Pacific		$19	$26
Prajna et al (2007)[84]	Corneal ulcers	India	2004USD	$86 ($76-95)	$108 ($96-119)
Murithi et al (2008)[42]	Ocular trauma	Kenya	2004 USD	$70 ($4-497)	$88 ($5-626)
Agarwal et al (2010)[80]	Cataract repair	Various Africa[c]	2007 USD	$340 ($90-1,100)	$390 ($103-1,262)
Gogate et al (2010)[81]	Cataract repair	India	2007 USD	$122-475	$140-545
Baltussen et al (2012)[56]	Trichiasis surgery	Sub-Saharan Africa	2005 Int. $	$81	$98
	Cataract repair			$107	$130
	Trichiasis surgery	Southeast Asia		$575	$698
	Cataract repair			$9	$11
Dave et al (2012)[109]	Laser treatment for retinopathy of prematurity	Peru	2010 USD	$2,496	$2,722
Gradin et al (2012)[82]	Cataract repair	Kenya	2009 USD	$274-344	$304-381
Evans et al (2014)[83]	Cataract repair	Malawi	2011 USD	$202	$214
		Zambia		$277	$293
**Orthopedics**					
Grimes et al (2014)[67]	Various orthopedic procedures	Malawi	2012 USD	$11-134	$11-138
Gurses et al (2003)[68]	Pediatric trauma: major surgical treatment	Turkey	1999 USD	$929 ($63-1,995)	$1,326 ($90-2,848)
	Pediatric trauma: minor procedures			$355 ($41-1,550)	$506 ($59-2,212)
Padhi et al (2007)[74]	Open tibial injuries	India	2006 USD	$100	$118
Gosselin et al (2008)[69]	Various orthopedic procedures	Cambodia	2006 USD	$117	$138
Dada et al (2009)[70]	Musculoskeletal injuries	Nigeria	2005 USD	$34-98	$41-119
Gosselin et al (2009)[75]	Femoral shaft fractures	Cambodia	2008 USD	$888-1,107	$981-1,223
Hsu et al (2009)[76]	Femoral fractures: elastic intramedullary nailing	Philippines	2006 USD	$844	$996
	Femoral fractures: dynamic skeletal traction spica casting			$216	$255
Reddy et al (2009)[71]	Various orthopedic injury procedures	India	2004 USD	$104	$130
Adegbehingbe et al (2010)[33]	Ponseti clubfoot management	Nigeria	2009 USD	$217	$240
Akinyoola et al (2011)[77]	Femoral shaft fractures	Nigeria	2007 USD	$51 ($14-190)	$59 ($16-218)
Gosselin et al (2011)[72]	Various orthopedic procedures	Nicaragua	2010 USD	$1,483	$1,612
		Dominican Republic		$1,483	$1,612
		Haiti		$1,201	$1,305
Chen et al (2012)[73]	Various orthopedic procedures	Nicaragua	2010 USD	$711	$773
Alinia et al (2013)[103]	Amputation	Iran	2009 USD	$3,471	$3,848
Wu et al (2013)[44]	Various orthopedic procedures	Kenya	2011 USD	$368	$388
Perveen et al (2014)[34]	Ponseti clubfoot management	Bangladesh	2011 USD	$38	$41
**Plastic and Reconstructive Surgery**					
Hodges et al (2000)[58]	Cleft lip & palate	Uganda	1999 British Pound	£27	$160
Nursal et al (2003)[100]	Various burn injuries	Turkey	2001 USD	$7,716	$10,361
Tollefson et al (2006)[59]	Cleft lip & palate	China	2004 USD	$1,590	$2,002
Corlew (2010)[60]	Cleft lip & palate	Nepal	2005 USD	$275	$334
Magee Jr. et al (2010)[61]	Cleft lip & palate	Vietnam	2008 USD	$86-230	$95-253
		Nicaragua		$631	$695
		Kenya		$432	$476
Allorto et al (2011)[101]	Various burn injuries	South Africa	2007 S. African Rand	R103,000-154,000	$28,425-42,499
Moon et al (2012)[64]	Cleft lip & palate	Vietnam	2010 USD	$335	$364
Alinia et al (2013)[103]	Burns, first degree	Iran	2009 USD	$599	$664
	Burns, second degree			$1,486	$1,647
	Burns, third degree			$4,342	$4,813
Poenaru (2013)[65]	Cleft lip & palate	Africa	2010 USD	$758	$824
		Americas		$633	$688
		Europe		$446	$485
		Middle East		$867	$942
		Southeast Asia		$759	$825
		Western Pacific		$817	$888
Rattray et al (2013)[43]	Various reconstructive procedures	Cambodia	2012 USD	$480	$496
Wu et al (2013)[44]	Various reconstructive procedures	Kenya	2011 USD	$384	$405
Zhu et al (2013)[102]	Various burn injuries	China	2010 Chinese Yuan	¥4,253	$1,354

	Intervention	Location	Currency	Cost per outcome, as reported[a]	Cost per outcome, 2015 USD[b]
**Urology**					
Manji (2000)[46]	Circumcision	Tanzania	1998 USD	$15-20	$22-29
Ekenze et al (2007)[41]	Genital reconstruction	Nigeria	2005 USD	$120 ($100-150)	$146 ($122-183)
Gray et al (2007)[47]	Circumcision	Uganda	2006 USD	$69	$81
Binagwaho et al (2010)[45]	Circumcision	Rwanda	2008 USD	$15	$17
Njeuhmeli et al (2011)[48]	Circumcision	Botswana	2010 USD	$78 ($62-94)	$85 ($68-102)
		Lesotho		$84 ($67-101)	$91 ($73-110)
		Malawi		$84 ($67-101)	$91 ($73-110)
		Mozambique		$86 ($69-104)	$94 ($75-113)
		Namibia		$87 ($69-104)	$94 ($76-113)
		Kenya		$75 ($60-90)	$82 ($65-98)
		Rwanda		$80 ($64-96)	$87 ($70-105)
		South Africa		$95 ($76-114)	$104 ($83-125)
		Swaziland		$75 ($60-90)	$82 ($65-98)
		Tanzania		$83 ($66-99)	$90 ($72-108)
		Uganda		$66 ($53-79)	$72 ($57-86)
		Zambia		$90 ($72-108)	$98 ($78-117)
		Zimbabwe		$78 ($63-94)	$85 ($68-102)
Tumwesigyea et al (2013)[49]	Circumcision	Uganda	2009 USD	$22	$24
Wu et al (2013)[44]	Various urologic operations	Kenya	2011 USD	$713	$751

Abbreviations include: ENT ear, nose & throat; DALY disability-adjusted life-year; LYS life-year saved; LYG life-years gained; HALY health-adjusted QALY quality-adjusted life-year; USD United States dollar; Int. $ International dollars (a hypothetical currency unit with the same purchasing power U.S. dollar has in the United States at a given time).

[a] Values converted to a per-procedure basis using information presented in article if not reported as such.

[b] Values converted to USD using purchasing power parity conversion factors from the World Bank[16] and inflated to 2015 USD using the Consumer Price calculator.[17]

[c] Includes South Africa, Malawi, Tanzania, Kenya, Uganda, Ethiopia, Sudan, Nigeria, Angola, Ghana, and Mali.
