# Supplementary material for: Economic Analysis of Children’s Surgical Care in Low- and Middle-Income Countries: A Systematic Review and Analysis
Source: PLoS One. 2016 Oct 28;11(10):e0165480. doi: 10.1371/journal.pone.0165480 (PMC5085034; doi:10.1371/journal.pone.0165480)
Supplement: S4 Table — (PDF) [file pone.0165480.s005.pdf]

S4 Table Health Outcomes and Societal Economic Benefit of Pediatric Surgical Procedures

	Intervention	Location	Unit of outcome	Outcome per procedure	Economic benefit per procedure (published or *calculated*)[a]
**Cardiac Surgery**					
Costa et al (2014)[94]	Atrial septal defects: percutaneous closure	Brazil	QALY	19.74	*$313,866*
	Atrial septal defects: surgical closure		QALY	19.71	*$313,389*
**ENT**					
Wu et al (2013)[44]	Various ENT procedures	Kenya	DALY	3-7.5	*$8,670-21,675*
**General Surgery**					
Jha et al (1998)[29]	Appendectomy	Guinea	LYS	1.86	*$2,120*
	Inguinal hernia repair		LYS	0.71	*$814*
Gosselin et al (2006)[26]	Various general surgery	Sierra Leone	DALY	1.31	*$2,401*
Shillcutt et al (2010)[36]	Inguinal hernia repair	Ghana	DALY	9.32-15.29	*$36,907-60,548*
Wang et al (2012)[97]	Cystic echinococcosis	China	DALY	1.03	$1,436
Ilbawi et al (2013)[50]	Various general surgery	Cameroon	DALY	7.9	*$23,226*
Shillcutt et al (2013)[38]	Inguinal hernia repair	Ecuador	DALY	6.39-10.79	*$71,057-119,985*
Wu et al (2013)[44]	Various general surgery	Kenya	DALY	10-24	*$28,900-69,360*
Eeson et al (2015)[39]	Inguinal hernia repair	Uganda	DALY	5.7-22	*$9,633-37,180*
**Neurosurgery**					
Warf et al (2011)[66]	Hydrocephalus	Uganda	DALY	11.74-19.94	$10,438-17,508
Wu et al (2013)[44]	Various neurological operations	Kenya	DALY	9-24	*$26,010-69,360*
Davis et al (2014)[57]	Various neurological operations	Guatemala	DALY	8.12	*$58,977*
**Ophthalmology**					
Baltussen et al (2005)[55]	Trichiasis surgery	Africa	DALY	1.43	*$5,650*
		Americas	DALY	1.43	*$1,589*
		Mediterranean	DALY	1.43	*$18,337*
		Southeast Asia	DALY	1.43	*$7,753*
		Western Pacific	DALY	1.43	*$18,160*
**Orthopedics**					
Grimes et al (2014)[67]	Various orthopedic procedures	Malawi	DALY	1.4	*$1,091*
Gosselin et al (2008)[69]	Various orthopedic procedures	Cambodia	DALY	4.23	*$13,029*
Gosselin et al (2011)[72]	Various orthopedic procedures	Nicaragua	DALY	4.1	*$35,118*
		Dominican Republic	DALY	4.1	*$19,159*
		Haiti	DALY	3.51	*$6,134*
Chen et al (2012)[73]	Various orthopedic procedures	Nicaragua	DALY	0.99-2.64	*$4,623-12,329*
Wu et al (2013)[44]	Various orthopedic procedures	Kenya	DALY	5-11	*$14,450-31,790*
**Plastic and Reconstructive Surgery**					
Corlew (2010)[60]	Cleft lip & palate	Nepal	DALY	2.5-16.6	$6,050-40,172
Magee Jr. et al (2010)[61]	Cleft lip & palate	Vietnam	DALY	0.27-11.63	*$1,445-62,221*
		Nicaragua	DALY	0.35-9.55	*$1,611-44,599*
		Kenya	DALY	0.36-4.50	*$1,040-13,005*
Alkire et al (2011)[62]	Cleft lip & palate	Sub-Saharan Africa	DALY	3.33-7.42	$7,257-12,718
Hughes et al (2012)[63]	Cleft lip & palate	Ecuador	DALY	3.88-10.22	$24,357-66,100
Moon et al (2012)[64]	Cleft lip & palate	Vietnam	DALY	4.57-6.54	*$24,450-34,989*
Poenaru (2013)[65]	Cleft lip & palate	Africa	DALY	3.8-9	*$15,048-35,640*
		Americas	DALY	3.8-9	*$113,696-269,279*
		Europe	DALY	3.8-9	*$70,478-166,920*
		Middle East	DALY	3.8-9	*$48,842-115,679*
		Southeast Asia	DALY	3.8-9	*$20,651-48,910*
		Western Pacific	DALY	3.8-9	*$57,544-136,287*
Rattray et al (2013)[43]	Various reconstructive procedures	Cambodia	DALY	5.2	*$16,029*
Wu et al (2013)[44]	Various reconstructive procedures	Kenya	DALY	5-11.5	*$14,450-33,235*
**Urology**					
Binagwaho et al (2010)[45]	Circumcision	Rwanda	LYG	0.04	*$69*
Wu et al (2013)[44]	Various urologic operations	Kenya	DALY	6-13	*$17,340-37,570*

Abbreviations include: ENT ear, nose & throat; DALY disability-adjusted life-year; LYS life-year saved; LYG life-year QALY quality-adjusted life-year; GDP gross domestic product;
[a] Values in italics were calculated using the human capital approach by multiplying the upper and lower bound estimates the most recently published PPP GNI/capita estimates obtained from the World Bank.[24]
